# Clinical Guidelines and New Molecular Targets for Cutaneous Lymphomas

**DOI:** 10.3390/ijms222011079

**Published:** 2021-10-14

**Authors:** Makoto Sugaya

**Affiliations:** Department of Dermatology, International University of Health and Welfare, Chiba 286-8520, Japan; sugayamder@iuhw.ac.jp

**Keywords:** mycosis funogides, Sézary syndrome, clinical guidelines, surface molecules, signaling pathway

## Abstract

Primary cutaneous lymphomas are heterogenous lymphoproliferative disorders. Some patients show rapid progression and the need for treatment of advanced disease is still unmet. The frequency of each subtype of cutaneous lymphoma varies among different ethnic groups, as do the medical systems found in different countries. It is important to know the differences in clinical guidelines in different areas of the world. Although current monochemotherapy with gemcitabine or pegylated liposomal doxorubicin is temporarily effective for mycosis funogides (MF) and Sézary syndrome (SS)—representative types of cutaneous lymphomas—the duration of response is usually limited. Therefore, treatment strategies targeting tumor-specific molecules have been developed. Molecular targets for MS/SS are currently CD30, CCR4, CD25, CD52, and histone deacetylases, most of which are surface molecules specifically expressed on tumor cells. As a result of advances in research techniques, different kinds of genomic alterations in MF/SS have been revealed. Molecular targets for MS/SS in the near future would be CD158k, JAK, PIK3, the mammalian target of rapamycin, and microRNAs, most of which mediate intracellular signaling pathways. Personalized therapy based on the detection of the genetic signatures of tumors and inhibition of the most suitable target molecules constitutes a future treatment strategy for MF/SS.

## 1. Introduction

Primary cutaneous lymphomas, a member of the class of non-Hodgkin lymphomas, are the most common group of extranodal lymphomas except for those which occur in the gastrointestinal tract. Patients with primary cutaneous lymphomas present with skin lesions without extracutaneous involvement of the disease at the time of diagnosis, although some have swollen superficial lymph nodes or blood involvement. Cutaneous lymphomas are heterogenous lymphoproliferative disorders, and their prognoses and treatments differ widely across cases. In order to decide treatment strategies based on prognostic expectations, a correct diagnosis on the basis of clinical images and histological and immunophenotypic findings is essential. Although the prognosis of most cutaneous lymphomas is excellent, some patients show rapid progression, and treatment of advanced disease is still an unmet medical need. In this article, clinical guidelines for the management of cutaneous lymphomas from different areas of the world were reviewed. In addition, current and future molecular targets for cutaneous lymphomas are discussed, with a special focus on mycosis funogides (MF) and Sézary syndrome (SS).

## 2. Classification of Cutaneous Lymphomas

The World Health Organization (WHO) classification of tumors of hematopoietic and lymphoid tissues was revised in 2016 [1] and published as the WHO Blue Book in 2017. In the following year, the WHO-European Organization for Research and Treatment of Cancer (EORTC) classification for primary cutaneous lymphomas was updated [2]. In the most recent classification, two types of cutaneous lymphomas have been added as provisional entities: (1) chronic EBV-positive mucocutaneous ulcer and (2) primary cutaneous acral CD8^+^ T cell lymphoma. Both subtypes of cutaneous lymphomas exhibit an excellent prognosis with characteristic clinical, histological, and phenotypic features [3,4]. It is important not to apply invasive treatment to patients with these lymphomas. In most cases of chronic EBV-positive mucocutaneous ulcer, discontinuance of immunosuppressive drugs, such as methotrexate, is enough. For primary cutaneous acral CD8^+^ T cell lymphoma, surgical resection can usually lead to complete remission. Primary cutaneous CD4^+^ small/medium lymphoma was renamed primary cutaneous CD4^+^ small/medium lymphoproliferative disorder because the prognosis has been revealed to be excellent.

Among various subtypes of cutaneous lymphomas, MF is the most common form, covering about half of all cutaneous lymphomas [5]. Skin lesions seen in MF patients are erythematous patches, plaques, or tumors. Most patients remain at the patch stage (early stage), while some progress to tumor stage (advanced stage) during a long clinical course [6], which usually takes more than ten years. SS is charcterized by erythroderma and lymph node and peripheral blood involvement. Tumor cells in both MF and SS are typically CD4^+^CCR4^+^ memory T cells [7] and some MF cases develop into SS. MF and SS are included in the same staging system in the current classification [8]. It was reported that MF and SS might arise from different types of memory T cells; MF from resident memory T cells and SS from central memory T cells [9]. The latter express L-selectin and CCR7, having the ability to move around between skin, lymph nodes and blood. Although the clinical and pathological features of typical MF and typical SS are clearly different, there are cases having characteristics of both diseases. Erythrodermic MF presents with erythroderma with lymph node swellings, without blood involvement. SS patients sometimes show MF-like tumors rather than erythroderma after initial treatment. It has been recently shown that tumor cells in SS and MF can have phenotypic features of any of the major naïve/memory T cell subsets, and phenotypic shifts within these subsets are frequently seen, suggesting that surface molecules on neoplastic T cells represent a functional status rather than the origin of cells [10]. The recent analysis using whole exome sequencing has revealed that the mutational burden of ultraviolet (UV) signature, which is rich in MF samples, is detected in tumor cells in the blood of SS patients, suggesting extensive re-circulation of the tumor cell through the skin and blood [11]. Therefore, the difference in origins of tumor cells in MF and SS is still a matter of controversy.

## 3. Guidelines for Cutaneous Lymphomas

### 3.1. Japanese Guidelines

There have been a number of guidelines on the management of cutaneous lymphomas published in Europe and North America. The frequency of each subtype of cutaneous lymphoma varies among different ethnic groups and there are many points of difference in the medical systems across countries. Not all drugs that can be used in the United States are available in the rest of the world. Skin color can have influence on both the effects and the side effects of treatments, such as UV phototherapy. Therefore, it is important for every country to have its own treatment guidelines. The first Japanese guidelines for the management of cutaneous lymphomas were published in 2009, and were updated as the second version in 2011 [12]. Since then, the WHO classification of tumors of hematopoietic and lymphoid tissues was revised in 2016 [1] and the WHO-EORTC classification for primary cutaneous lymphomas was updated in 2018 [2]. Moreover, some new drugs for cutaneous T cell lymphoma (CTCL) have been approved in Japan. Therefore, the Japanese guidelines for cutaneous lymphoma were updated in 2019 and their English version was published in 2020 [13]. Clinical questions (CQs) and recommendations in accordance with the Grading of Recommendations, Assessment, Development and Evaluation scheme, which are essential for recent guidelines, have been described in the latter half of the guidelines. Recommendations for CQs were made after long discussion by panel members.

### 3.2. British Guidelines

The British Association of Dermatologists published guidelines for cutaneous lymphomas in 2019 [14]. Compared with Japanese guidelines, many topical treatments, such as mechlorethamine, bexarotene gel, and carmustine, are described, probably because a long-term treatment with UV phototherapy is not practical for Caucasian patients. They have clearly denied the benefit of UV as a maintenance therapy because of a high risk of developing skin cancers, which is rare in Japanese. Interferon-α is used to treat CTCL in Britain, while interferon-γ is approved in Japan. Extracorporeal phototherapy is recommended for erythrodermic patients, which we cannot use in Japan. Histone deacetylase (HDAC) inhibitors, on the other hand, cannot be used in Britain. The importance of radiation therapy for B cell lymphoma is emphasized. Recommendations based on a literature review are described, while CQs are not provided.

### 3.3. European Guidelines

EORTC and the European Society for Medical Oncology (ESMO) published guidelines for treatment of cutaneous lymphomas in 2017 and in 2018, respectively [15,16]. EORTC guidelines are a kind of textbook for the treatment of MF and SS. Evidence levels fot treatment options are described, while CQs are not set up. EMSO guidelines are basically the same as the British guidelines, but the details in each treatment modality are described. The recommendation levels for therapies seem to be rather high considering the paucity of evidence. There are no CQs in the EMSO guidelines.

### 3.4. U.S. Guidelines

National Comprehensive Cancer Network guidelines for the management of cutaneous lymphomas are shown as slides. Each slide contains too much information for beginners of the field. Treatment algorithms are useful, although almost all of them are composed of multiple slides, making them difficult to follow. Many treatment options are described and are summarized in several categories. Comparisons between each treatment are not made, nor are CQs described. Almost all treatments available worldwide are described and there is a large number of references, meaning that the guidelines can be used as an encyclopedia. The similarities and differences between clinical guidelines are summarized in Table 1.

## 4. Current Therapeutic Target Molecules

**Figure 1:**
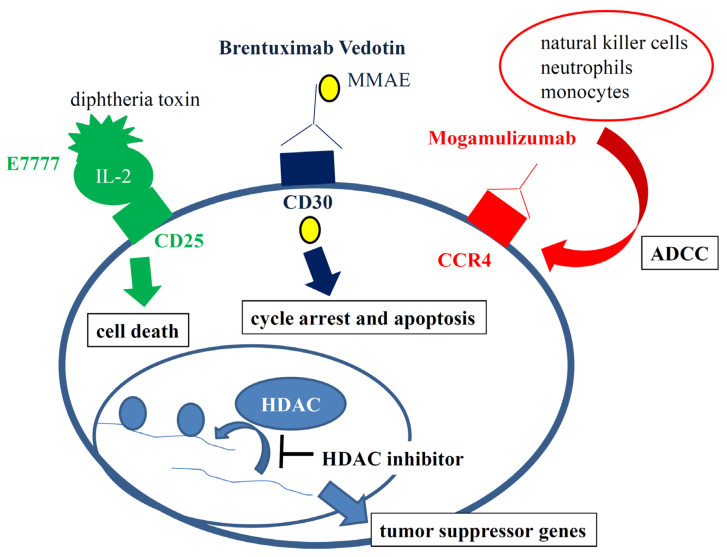
Current molecular targets for MS/SS are CD30, CCR4, CD25, CD52, and HDACs. Brentuximab Vedotin is an antibody–drug conjugate made of a chimeric monoclonal anti-CD30 antibody and monomethyl auristatin E (MMAE), a cytotoxic antitubulin agent. Mogamulizumab is a humanized monoclonal antibody against CCR4, whose antitumor activity is mediated by antibody-dependent cellular cytotoxicity (ADCC). E7777 is a sophisticated version of denileukin diftitox, which is a fusion protein composed of human IL-2 and the cytotoxic and membrane-translocating domains of the diphtheria toxin. Histone deacetylase (HDAC) inhibitors increase acetylation of histones and other proteins, leading to the remodeling of chromatins and an increase in the expression of tumor suppressor genes.

Treatment of advanced MF/SS is still a challenge. Although monochemotherapy with gemcitabine or pegylated liposomal doxorubicin is temporarily effective, the duration of response is usually limited. Allogeneic hematopoietic stem cell transplantation is the only curative treatment option, although the treatment-associated mortality is high and relapses are not rare. Even if there is no recurrence, graft versus host diseases and various kinds of infections can be fatal. Therefore, treatment strategies targeting tumor-specific molecules have been developed. Molecular targets for MS/SS are currently CD30, CCR4, CD25, CD52, and HDACs (Figure 1).

### 4.1. CD30

In some MF/SS cases, tumor cells express CD30, which belongs to the tumor necrosis factor receptor family. Not only tumor cells found in MF/SS—primary cutaneous anaplastic large cell lymphoma (pcALCL) and Hodgkin lymphoma—but also those in adult T cell leukemia/lymphoma (ATL) and other types of cutaneous lymphomas can express this molecule on the surface. Brentuximab Vedotin (BV) is an antibody–drug conjugate made of a chimeric monoclonal anti-CD30 antibody and monomethyl auristatin E (MMAE), a cytotoxic antitubulin agent [17]. This drug targets CD30^+^ cells, which internalize BV, resulting in the release of MMAE. Inhibition of tubulin polymerization induced by MMAE can cause cell cycle arrest and apoptosis. MMAE, released from CD30^+^ cells, can kill neighboring cells. The international randomized phase 3 trial (ALCANZA study) for pretreated CD30^+^ MF or pcALCL showed that BV had a superior effect on the overall response rate (ORR), lasting at least 4 months against the standard therapies chosen by physicians—methotrexate (5–50 mg weekly) or bexarotene (300 mg/m^2^ daily) [18]. The most frequent adverse event (AE) was peripheral neuropathy, observed in two thirds of BV-treated patients. In 2017, BV was approved for the treatment of adult patients with CD30^+^ MF and pcALCL who have received prior systemic therapy by the US Food and Drug Administration (FDA) and the European Medicines Agency. By contrast, BV is only approved for the treatment of CD30^+^ peripheral T cell lymphoma (PTCL), systemic ALCL, and Hodgkin lymphoma in Japan. As the effect of the drug is not mediated by antibody-dependent cellular cytotoxicity (ADCC), it can be effective for patients whose residual immune function is not high. Neuralgia, an unfortunate side effect, occurred in about 60% of patients [18,19]. Among the 33 patients who experienced the side effect who had systemic ALCL, 91% experienced resolution or improvement within two years [19]. Judging from its high efficacy, BV should be considered first when tumor cells express CD30.

### 4.2. CCR4

CCR4 is expressed not only by type 2 helper T cells and regulatory T cells but also by tumor cells of MF, SS, and ATL [7,20]. Mogamulizumab is a humanized monoclonal antibody against CCR4, whose antitumor activity is mediated by ADCC. Mogamulizumab was approved in Japan for relapsed or refractory CCR4^+^ ATL in 2012, and for PTCL and CTCL in 2014. A multicenter phase II study for patients with relapsed PTCL and CTCL had been performed before mogamulizumab was approved for use in Japan. In the study, seven patients with MF were included [21]. The ORR for MF patients was 28.6%, with no complete response (CR). In the US, 38 patients with pretreated MF/SS were enrolled in a phase I/II study [22]. The ORR for SS (*n* = 17) was 47.1% and that for MF (*n* = 21) was 28.6%, suggesting that mogamulizumab was more effective as a treatment for SS than for MF. In the phase 3 multicentric clinical trial for relapsed or refractory MF/SS (MAVORIC study), mogamulizumab showed a significantly better effect in progression free survival than the HDAC inhibitor vorinostat [23]. Considering that tissue-specific response rates were 42% in skin, 68% in blood, 17% in lymph nodes, and 0% in viscera, mogamulizumab seems to be effective especially for tumor cells in the blood, probably because many natural killer cells, neutrophils, or monocytes, which are important for ADCC, are intact and accessible to tumor cells. In all studies, common AEs were nausea, fever, pruritus, lymphopenia, skin eruptions, and infusion reactions. In 2018, the FDA and European Medicines Agency approved mogamulizumab for the treatment of patients with CTCL who have received at least one prior systemic therapy. As mogamulizumab is dependent on ADCC for its effect, the drug is not so effective when the tumor burden is rapidly increasing. In that case, introduction of mogamulizumab after irradiation is a good combination strategy. CCR4 is also expressed by regulatory T cells. Depletion of regulatory T cells by this drug after irradiation may lead to a long remission. We cannot be too careful in considering the use of this drug for patients who may be candidates for allogeneic hematopoietic stem cell transplantation in the near future, as prior treatment with mogamulizumab may induce severe acute graft-versus-host disease, which was reported in ATL patients [24]. Mogamulizumab is shown to be more effective in ATL patients with CCR4 mutation [25]. It would be very useful to know whether the same is true in cases of MF/SS.

### 4.3. CD25

The IL-2 receptor (IL-2R) consists of three forms with low, intermediate, and high affinity. CD25 is the IL-2R α chain, expressed by tumor cells in most cases of ATL and pcALCL and in some cases of MF/SS. This molecule is also expressed on the surface of regulatory T cells. Denileukin diftitox is a fusion protein composed of human IL-2 and the cytotoxic and membrane-translocating domains of the diphtheria toxin. After binding to the IL-2R on tumor cells, this drug is internalized and the diphtheria toxin inhibits protein synthesis, leading to cell death [26]. This drug is supposed to kill not only CD25-expressing tumor cells but also regulatory T cells, which may result in enhancement of tumor immunity. In a multicenter, randomized, double-blind, placebo-controlled phase III trial, including 144 stage IA–III pretreated MF/SS patients, the ORR was 44% for all denileukin diftitox-treated patients, with 10% CR and 34% partial response (PR) [27]. The main AEs were infusion reaction and capillary leak syndrome. The FDA approved denileukin diftitox in 2008 for the treatment of patients with persistent or recurrent CD25^+^ CTCL. E7777 is a sophisticated version of denileukin diftitox, having improved purity and an increased percentage of active monomer. In a multicenter, single-arm phase II study of E7777 for patients with relapsed or refractory PTCL (17 cases) and CTCL (19 cases) in Japan, the ORR was 36% (41% for PTCL and 31% for CTCL) [28]. Although there was no statistical significance, cases with higher expression levels of CD25 on tumor cells tended to respond better to the drug, which was also true for denileukin diftitox [29]. The drug is now approved in Japan for relapsed or refractory PTCL and CTCL. Similar to BV, this drug does not depend on ADCC, making it possible to be effective in advanced stages.

### 4.4. CD52

CD52 is expressed on different types of immune cells, including normal and malignant T cells. A humanized IgG1 antibody targeting the CD52 antigen is alemtuzumab. In the phase 2 clinical study of alemtuzumab for advanced MF/SS, the ORR was 55%, with 32% CR and 23% PR [30]. The drug was especially effective for erythrodermic patients (69% ORR, with 38% CR). Severe AEs, such as infusion reaction, hematologic toxicities, and infectious complications, in the previous trial led to another clinical trial of alemtuzumab at a low dose for SS [31]. The ORR was similar to that for a high dose of alemtuzumab, with decreased AEs. Even though alemtuzumab can be promising for SS, the drug is currently only available through compassionate use programs for hematological malignancies in the US and Europe. In Japan, alemtuzumab is not approved for CTCL.

### 4.5. HDAC

Histone acetylation and deacetylation are essential parts of gene regulation, which is mediated by histone acetyltransferase and HDAC. Acetylation of histone decreases the interaction of the N termini of histones with DNA, inducing greater levels of gene transcription [32]. HDAC inhibitors increase acetylation of histones and other proteins, leading to the remodeling of chromatins and an increase in expression of tumor suppressor genes. Thus, the drugs can induce apoptosis of target cells. Common AEs by HDAC inhibitors are fatigue, thrombocytopenia, diarrhea, and nausea [33].

Vorinostat, the first approved HDAC inhibitor, is an oral drug that can competitively inhibit class I/II HDAC enzymes. In the phase 2B multicenter trial with 74 stage IB-IVA MF/SS patients, the ORR was 29.7% [34]. Drug-related AEs were diarrhea, fatigue, nausea, thrombocytopenia, hyperbilirubinemia, and anorexia. Vorinostat was approved by the FDA for the treatment of CTCL patients with two previous systemic therapies in 2006. The drug was approved in 2011 in Japan based on the phase 1 clinical trial [35].

Romidepsin is a bicyclic peptide that selectively inhibits class I HDAC. In an international single-arm open-label phase II study for 96 stage IB–IVA CTCL patients, the ORR was 34% [36]. Interestingly, the ORR in MF patients with tumors was 45% (9/20) and that in patients with folliculotropic MF was 60% (6/10), which tended to be higher than for other patients [37]. In 2009, the FDA approved romidepsin for the treatment of CTCL. In Japan, romidepsin is now approved for peripheral T cell lymphoma, but not for CTCL.

Belinostat is a pan-HDAC inhibitor that should be given by an intravenous infusion. The FDA approved the drug for the treatment of relapsed or refractory PTCL in 2014. In the phase 2 clinical trial for relapsed or refractory PTCL and CTCL, including 29 patients with CTCL (17 MF and seven SS), the ORR was 13.8% (10.3% CR and 3.4% PR) [38]. Panobinostat is an oral drug that can inhibit all HDACs. In 2015, the FDA approved it for the treatment of multiple myeloma. In a phase 2 study including 139 patients with stage IB-IVA MF/SS, the ORR was 17.3% [39].

## 5. Future Therapeutic Target Molecules

**Figure 2:**
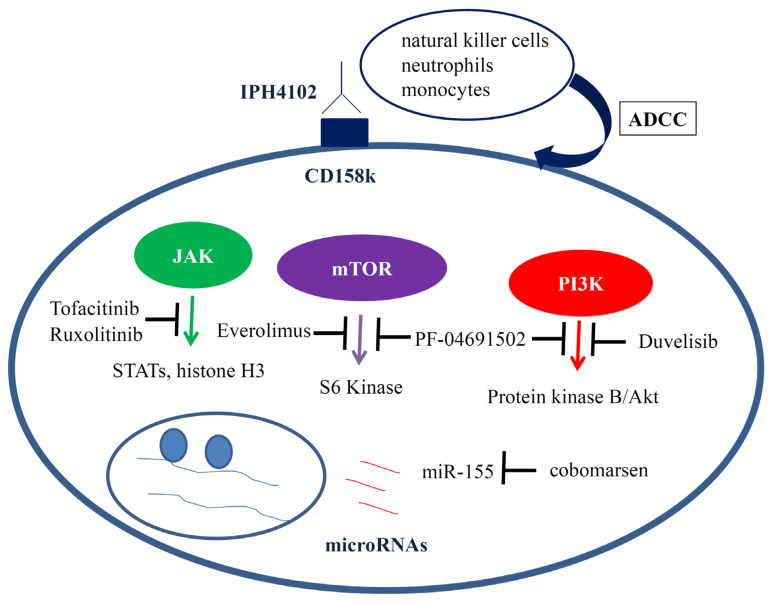
Molecular targets for MS/SS in the near future would be CD158k, JAK, phosphoinositide-3-kinase (PI3K), the mammalian target of rapamycin (mTOR), and microRNAs. IPH4102, a humanized, monoclonal antibody against CD158k, can selectively and efficiently deplete primary Sézary cells through antibody-dependent cell cytotoxicity (ADCC) and phagocytosis. Ruxolitinib, a JAK1/2 inhibitor, and Tofacitinib, a JAK 1/3 inhibitor, exhibit anti-tumor effects in CTCL cell lines in vitro. Duvelisib, an oral drug that can inhibit both PI3K-δ and PI3K-γ, has both experimental and clinical activity. Everolimus, an oral agent that targets the mTOR pathway, has clinical effect on relapsed or refractory T cell lymphomas. PF-04691502 is a recently developed drug that can inhibit both mTOR and PI3K pathways. Cobomarsen, a locked nucleic acid-modified oligonucleotide inhibitor of *miR-155*, inhibits *miR-155* expression, reduces cell survival signaling, decreases cell proliferation, and induces apoptosis.

As a consequence of advances in research techniques, we can analyze lesional skin of MF/SS patients even at the single-cell level. Whole exome/genome sequencing studies of MF/SS patients have suggested that somatic mutations in genes involved in TCR/NFκB signaling, Th2 differentiation, apoptosis, chromatin modifying genes, and cell-cycle control are important for development of the disease [40,41]. A wide variety of genomic alterations have been detected in MF/SS and the genetic signature of tumors are different in different patients. No specific genomic alterations or molecular abnormalities can differentiate MF/SS from other lymphomas. Significant molecular heterogeneity in gene expression between different patients and even within the same patients over time was also reported on the basis of transcription expression profiling [42]. Moreover, a high degree of single-cell heterogeneity within the malignant T cell population in MF/SS has been reported [43,44,45]. Personalized therapy based on detection of the genetic signature of tumors and inhibition of the most suitable target molecules constitutes a future treatment strategy for MF/SS. Molecular targets for MS/SS in the near future would be CD158k, JAK, PIK3, the mammalian target of rapamycin (mTOR), and microRNAs (Figure 2).

### 5.1. CD158k

CD158k, also called KIR3DL2, belongs to the highly polymorphic killer cell immunoglobulin-like receptor family. Certain subsets of normal CD8^+^ T cells and NK cells express this molecule. By contrast, normal CD4^+^ cells do not express it. It has been revealed that CD158k is expressed by tumor cells in SS, advanced MF, and pcALCL [46,47]. IPH4102, a humanized, monoclonal antibody against CD158k, can selectively and efficiently deplete primary Sézary cells through ADCC and phagocytosis [48]. In an international open-label phase 1 clinical trial, 44 patients with relapsed or refractory CTCL, including 35 (80%) patients with SS, eight (18%) with MF, and one (2%) with CTCL, not otherwise specified, were enrolled [49]. In the dose-escalation part, no dose limiting toxicity was found. The most common AEs were peripheral edema and fatigue, all of which were grade 1–2. The ORR was 36.4%, while it was 43% in SS patients. Therefore, IPH4102 is a promising drug for treating patients with relapsed or refractory CTCL, particularly those with SS.

### 5.2. JAK

In some cases of MF/SS, activating mutations in the JAK/STAT pathway, including *JAK1*, *JAK3*, *STAT3*, and *STAT5B*, were reported [40,41,50]. Moreover, in MF, recurrent deletion of two tumor suppressor genes (*HNRNPK* and *SOCS1*), both of which inhibit JAK-STAT signaling, has been detected [51]. Therefore, the JAK/STAT pathway can be a therapeutic target for those patients. Ruxolitinib, a JAK1/2 inhibitor, in combination with the HDAC inhibitor resminostat, showed substantial anti-tumor effects in two CTCL cell lines in vitro [52]. On the other hand, JAK3 is ectopically expressed in various CTCL cell lines and tumor cells in the blood of SS patients [53]. JAK3 induced tyrosine phosphorylation of recombinant human Histone H3, which was blocked by JAK 1/3 inhibitor Tofacitinib. Thus, JAK inhibitors have shown promising results, at least in in vitro models. Most new therapies for atopic dermatitis and psoriasis, including biologics like dupilumab, are contraindicated for CTCL. When JAK inhibitors, some of which are already approved for atopic dermatitis and psoriatic arthritis, are proved to be effective for CTCL, clinicians can feel safe using the drugs to treat patients whose diagnosis is difficult to arrive at.

### 5.3. PI3K

Phosphoinositide-3-kinase (PI3K), a lipid kinase, is an important protein in intracellular signal transduction which can regulate multiple steps in oncogenesis. The PI3K-δ and PI3K-γ isoforms are mainly expressed by leukocytes, mediating multiple pathways. These isoforms are essential for cell survival, proliferation, and differentiation. PI3K signaling is important not only for the proliferation of tumor cells but also for establishment of the tumor microenvironment through juxta-, para-, and endocrine effects on the surrounding cells. Moreover, PI3K-γ can inhibit phagocytosis by tumor-associated macrophages, suppressing antitumor immune response [54]. Therefore, inhibition of PI3K-δ and PI3K-γ is promising for the treatment of malignancies. Duvelisib, also called IPI-145, is an oral drug that can inhibit both PI3K-δ and PI3K-γ. A recent phase 1 open-label trial showed promising results, with the ORRs in patients with PTCL (*n* = 16) and CTCL (*n* = 19) being 50.0% and 31.6%, respectively [55]. Representative AEs were liver dysfunction, skin rash, and neutropenia. In vitro, duvelisib killed cell lines with constitutive phospho-AKT. In mouse xenograft models, after administration of duvelisib, M2 phenotypes among tumor-associated macrophages shifted to M1 phenotypes. Thus, duvelisib has shown both experimental and clinical activity.

### 5.4. mTOR

The mTOR pathway regulates cellular growth and metabolism. This molecule is involved in the pathogenesis of various cancers, which is also true for MS/SS. Rapamycin significantly suppressed tumor growth in a mouse model of T cell lymphoma using a marine T cell lymphoma line, MBL2, and human CTCL lines [56]. Everolimus is an oral agent that targets the mTOR pathway. A phase 2 clinical trial for 16 patients with relapsed or refractory T cell lymphoma was performed [57]. In the study, 7 patients with MF were enrolled. The ORR was 44% and 3 out of 7 MF patients showed PR. The most frequent AEs were hematologic toxicity and skin rash. Recently, the dual-mTOR/PI3K inhibitor PF-04691502 has been developed, showing promising results using in vitro models [58].

### 5.5. MicroRNAs

MicroRNAs are small non-coding RNA molecules which are usually composed of 20–25 nucleotides. They can directly degrade mRNA of target genes or bind to the 3′ untranslated region of targeted transcripts, repressing posttranscriptional protein translation. It has been reported that there are different kinds of microRNAs which have either tumor-promoting or -suppressing properties. The profile of microRNAs has been studied for the diagnosis and management of MF/SS [59]. Quantification of *miR-155*, *miR-203*, and *miR-205* by RT–PCR distinguished CTCL from benign disorders with high specificity and sensitivity [60]. Measuring plasma levels of microRNAs can also be used to detect CTCL [61]. Up-regulation of *miR-155* and down-regulation of *miR-203*/*miR-205* proved to be very useful. CTCL patients can be divided into high-risk and low-risk groups of disease progression based on expression levels of *miR-106b-5p*, *miR-148a-3p*, and *miR-338–3p* [62]. CD4^+^ neoplastic T cells from CTCL patients expressed a higher level of *miR-214* than healthy donors. When IL-15 transgenic mice with spontaneous *miR-214*-overexpressing CTCL were treated with *antagomiR-214*, disease severity was significantly decreased [63]. Treatment of CTCL cell lines with cobomarsen, a locked nucleic acid-modified oligonucleotide inhibitor of *miR-155*, inhibited *miR-155* expression, reduced cell survival signaling, decreased cell proliferation, and induced apoptosis [64]. A first-in-human phase 1 clinical trial of cobomarsen for CTCL patients is currently underway.

## 6. Conclusions

Although most patients with early CTCL show a good clinical course with a normal life expectancy, prognoses for advanced CTCL patients are very poor. Current treatment outcomes are not satisfactory, with high relapse rates and low durable remission rates. The current target molecules are mainly surface molecules specifically expressed on tumor cells. Although the antibodies against these molecules can be used for most patients, their efficacy may diminish when host immunity is impaired at advanced stage. Most of the future target molecules are mediators of intracellular signaling pathways. In general, these treatments are not so expensive as biologics targeting surface molecules. The most suitable target molecule should be decided for each individual patient, as the genetic signatures of tumors differ across patients.

## Figures and Tables

**Table 1:** The similarities and differences between the various sets of clinical guidelines.

	Japanese	British	EORTC	ESMO	NCCN
Year of update	2020	2018	2017	2018	2020
Target diseases	All PCL	All PCL	MF/SS	All PCL	All PCL
CQs	Yes	No	No	No	No
Treatment for MF/SS					
Mechlorethamine	No	Yes	Yes	Yes	Yes
Bexarotene gel	No	No	No	No	Yes
Carmustine	No	Yes	Yes	Yes	Yes
Interferon-α	N0	Yes	Yes	Yes	Yes
Interferon-γ	Yes	No	No	No	Yes
ECP	No	Yes	Yes	Yes	Yes
E7777	Yes	No	No	No	No
BV	No	Yes	No	Yes	Yes
Mogamulizumab	Yes	Yes	No	No	Yes
Alemtuzumab	No	No	No	No	No
Vorinostat	Yes	No	No	No	Yes
Romidepsin	No	No	No	No	Yes

PCL: primary cutaneous lymphoma; MF: mycosis fungoides; SS: Sézary syndrome; CQs: clinical questions; ECP: extracorporeal photopheresis; BV: brentuximab vedotin.

## Abbreviations

MF	mycosis funogides
SS	Sézary syndrome
WHO	World Health Organization
EORTC	European Organization for Research and Treatment of Cancer
UV	ultraviolet
CTCL	cutaneous T cell lymphoma
CQ	clinical question
HDAC	histone deacetylase
ESMO	European Society for Medical Oncology
pcALCL	primary cutaneous anaplastic large cell lymphoma
ATL	adult T cell leukemia/lymphoma
BV	brentuximab vedotin
MMAE	monomethyl auristatin E
ORR	overall response rate
AE	adverse event
FDA	Food and Drug Administration
PTCL	peripheral T cell lymphoma
ADCC	antibody-dependent cellular cytotoxicity
CR	complete response
IL-2R	IL-2 receptor
PR	partial response
mTOR	mammalian target of rapamycin
